# Role of the angiopoietin/Tie system in pregnancy (Review)

**DOI:** 10.3892/etm.2015.2280

**Published:** 2015-02-09

**Authors:** D. KAPPOU, S. SIFAKIS, A. KONSTANTINIDOU, N. PAPANTONIOU, D.A. SPANDIDOS

**Affiliations:** 1 First Department of Obstetrics and Gynecology, University of Athens, Athens, Greece; 2 Department of Obstetrics and Gynecology, University of Crete Medical School, Heraklion, Crete, Greece; 3 First Department of Pathology, University of Athens Medical School, Athens, Greece; 4 Third Department of Obstetrics and Gynecology, University of Athens, Athens, Greece; 5 Laboratory of Clinical Virology, University of Crete Medical School, Heraklion, Crete, Greece

**Keywords:** angiopoietin-1, angiopoietin-2, angiopoietin/Tie system, intrauterine growth restriction, placenta, preeclampsia, pregnancy, vascular endothelium-specific receptor tyrosine kinase Tie-2

## Abstract

Angiopoietin-1 and -2 are endogenous ligands for the vascular endothelium-specific receptor tyrosine kinase Tie-2. The angiopoietin/Tie system plays a critical role in the regulation of endothelial cell survival and vascular maturation and stability. Apart from its well-established role in vascular morphogenesis, emerging data support the involvement of angiopoietins in inflammation and various malignancies. Previous studies have underlined the significance of several angiogenic factors in normal placental development. In addition, angiogenic imbalance is observed in pregnancy complications related to impaired placentation, such as preeclampsia (PE) and intrauterine growth restriction (IUGR). However, there is only limited information available on the role of the angiopoietin/Tie system in the establishment of a competent feto-maternal vascular system. In this review, we present the current knowledge regarding the role of angiopoietins in normal pregnancy and pregnancy complications.

## 1. Introduction

Successful placentation is dependent on the establishment of a competent vascular network formed by two processes: vasculogenesis, which involves the *de novo* formation of vessels from endothelial progenitor cells and branching and non-branching angiogenesis, which is the remodeling of the pre-existing vessels (1). The imbalance between pro-angiogenic and anti-angiogenic factors can lead to impaired placentation, causing major pregnancy complications, such as preeclampsia (PE) and intrauterine growth restriction (IUGR), which can lead to poor obstetric outcomes (2,3). The role of various angiogenic factors in the pathophysiology of these conditions in pregnancy has been investigated (4). In recent years, in the field of angiogenesis research, studies have focused on the serum levels and placental expression of vascular endothelial growth factor (VEGF) and placental growth factor (PlGF) and its receptors in normal and pathological pregnancies (5,6). However, there is only limited information available regarding the role of the angiopoietin/Tie signaling system in gestation, that is a second vascular endothelium-specific receptor tyrosine kinase pathway apart from the VEGF system (7). Since there is emerging evidence of the involvement of angiopoietins in pathologies characterized by vascular remodeling (8–10), such as sepsis, diabetic retinopathy and various malignancies, it would be of interest to explore the significance of these factors in the establishment of a competent feto-maternal vascular system that is essential for proper placental function and fetal growth.

In this review, we present current evidence of the function of angiopoietins and provide a detailed description of available data on the role of the angiopoietin/Tie pathway in normal placental development and fetal growth, as well as in pregnancy complications related to defective placentation, such as PE and IUGR.

## 2. The angiopoietin/Tie system

**Figure 1:**
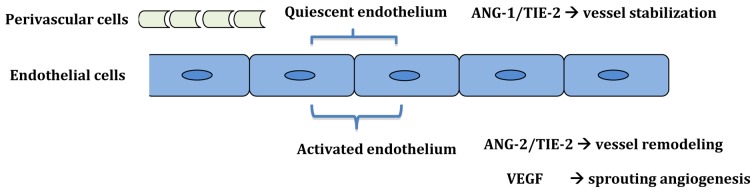
Role of the angiopoietin/Tie system in vessel remodeling and stabilization.

The angiopoietin system includes four ligands (Ang-1, Ang-2, Ang-3 and Ang-4), of which the most well characterized are Ang-1 and Ang-2, and two corresponding tyrosine kinase receptors (Tie-1 and Tie-2) (11,12). During pregnancy, angiopoietins are mainly produced by the placenta and seem to play a critical role in endothelial cell survival and the remodeling of vessels (Fig. 1). In particular, these factors seem to act complementary to the VEGF system and contribute to the later stages of angiogenesis (12). Both Ang-1 and Ang-2 bind to Tie-2, an endothelial cell-specific tyrosine kinase receptor with similar affinity (13,14). Although Ang-1 and Ang-2 share a similar protein structure (Ang-2 is 60% homologous to Ang-1), their biological activities differ significantly (13). Ang-1 acts as a paracrine agonist to Tie-2, leads to receptor dimerization and induces its phosphorylation on several cytoplasmic residues to activate downstream signaling pathways, including the phosphoinositide 3 (PI3)-kinase/Akt and extracellular signal-regulated kinase (ERK) pathways (15). The activation of the Akt pathway leads to the inhibition of FOXO transcription factors and downregulates the expression of Ang-2, endothelial cell-specific molecule 1 (ESM1) and PlGF (16). Apart from endothelial cells, previous studies have indicated that a distinct population of monocytes, known as Tie-2 expressing monocytes (TEM) and early hematopoietic cells also express the Tie-2 receptor (17,18). The other known Tie receptor (Tie-1; tyrosine kinase with immunoglobin and epidermal growth factor homology domains) seems to have weak kinase activity and its functional role has not yet been fully elucidated (19). Ang-1 promotes the reorganization of endothelial cells and the structural integrity of blood vessels by recruiting and interacting with peri-endothelial cells (14,19). An additional role of Ang-1 is to inhibit the activation of the vascular endothelial barrier and reduce the leakage and leucocyte migration into tissues induced by inflammatory agents (20). Despite the fact that the basic functioning of the pathway has been explored, there is no consistency as to the role of Ang-2 in certain conditions of pathological vascular remodeling, such as cancer and inflammation. Several lines of evidence suggest that Ang-2 binds to Tie-2, but primarily acts as an antagonist of Ang-1 signaling. In particular, Ang-2 disrupts the connections between the endothelium and perivascular cells and promotes cell death and vascular regression. In addition, Ang-2 renders endothelial cells more accessible to VEGF that promotes neovascularization (13,19,21). However, in the case of insufficient angiogenic stimuli, Ang-2 causes endothelial cell apoptosis and vessel regression (13).

Challenging this predominant view of the antagonistic Ang-1/Ang-2 concept, there are data suggesting that with longer exposure or at higher concentrations, Ang-2 acts as a Tie-2 agonist in cultured endothelial cells (22,23). In support of this view, in a previous study, when the Ang-2 gene was replaced with a cDNA encoding Ang-1 in Ang-2-deficient mice, the phenotype was partly rescued (24). Therefore, Ang-2 is currently considered as a context-dependent agonist or an antagonist of Tie-2. A recent study also stated that Ang-2 induced Tie-2-independent signaling by binding to integrins on endothelial cells that expressed low levels of Tie-2 and induced the phosphorylation of integrin adaptor protein, FAK, resulting in RAC1 activation (25). Furthermore, cellular experiments have demonstrated that Ang-2 modulates the responsiveness of endothelial cells to pro-inflammatory cytokines, such as tumor necrosis factor (TNF)-α and sensitizes these cells to an inflammatory response through an autocrine mechanism (26). Independently from their well-described role in the vascular endothelium, angiopoietins are also involved in the migration and proliferation of trophoblasts and the regulation of nitric oxide release during placentation (27).

Research interest has mainly been focused on the involvement of angiopoietins in various malignancies (28). In particular, the altered expression of Ang-1 and Ang-2 has been observed in several types of cancer, including ovarian and breast cancer, gastric carcinoma, melanoma and non-small cell lung cancer (28–30). In addition, recent studies producing encouraging results have suggested the potential use of specific Ang-2 inhibitors as anti-angiogenic agents to target the tumor vasculature, impairing its development and growth (31,32). There is also accumulating evidence indicating that serum Ang-2 may also be a useful tool as a candidate prognostic biomarker for patients with certain types of cancer, such as colorectal cancer and melanoma (33,34). The exact role of angiopoietins has also been broadly examined in other pathological conditions characterized by extensive vascular remodeling, such as diabetic retinopathy and sepsis (8,10).

## 3. The role of angiopoietins in normal pregnancy

The first step towards the understanding of the mechanisms through which angiopoietins contribute to the development of pathological conditions in pregnancy is to explore the role of these molecules in normal pregnancy. Ang-1 and Ang-2 are both expressed in the placenta from the very early stages of pregnancy and they mediate a number of endothelial and non-endothelial effects that are thought to be pivotal for proper placental development (27,35,36). Throughout gestation, the placental expression of Ang-1 normally increases, whereas that of Ang-2 and Tie-2 decreases (35,36). The same trend has been demonstrated for the serum levels of these factors (37). The significance of these molecules in early placentation is also highlighted by the fact that the mRNA expression of Ang-2 during the first trimester was found to be 400-fold higher than Ang-1 mRNA expression and 100-fold higher than VEGF mRNA expression, a factor which has an established role in placental angiogenesis (35). A possible explanation for the predominance of Ang-2 mRNA expression over that of the two angiogenic factors is that Ang-2 likely permits the fetal blood vessels to undergo remodeling and restructures the placenta vasculature to meet the increased oxygen and metabolic demands of the growing fetus. In addition, a previous cross-sectional study on 20 normal pregnancies at 8–41 weeks reported that the placental and protein expression of Ang-2 highly correlated and that they both markedly decreased toward term (35). During pregnancy, the placenta is most probably the main source of circulating Ang-2 as Ang-2 serum levels decrease rapidly following delivery (38).

A few previous *in situ*-hybridization studies have described the localization pattern of angiopoietins in the placenta in different stages of pregnancy. Seval *et al* (2008) demonstrated that in the very early human placenta (as early as the 4th week), Ang-1 protein was localized only in the syncytiotrophoblast, while Ang-2 was localized primarily in the syncytiotrophoblast, and to a lesser extent in the cytotrophoblastic layers of the placental villi (39). This is consistent with a previous report by Zhang *et al* (2001), who demonstrated that Ang-2 mRNA expression was readily detected in the syncytiotrophoblast in first trimester placenta (36). In the human placenta, high levels of Ang-2 mRNA expression have also been observed in perivascular cells and stromal macrophages in immature intermediate villi during the first and second trimester and in mature intermediate and terminal villi during the third trimester (35). Ang-1 is also weakly expressed in endothelial cells within intermediate villi, which is consistent with its role in promoting vascular maturation and stabilization (39). In a previous study, Dunk *et al* (2000) demonstrated that Ang-1 and Tie-2 were detected in the trophoblast bilayer of a first-trimester placenta, whereas Ang-2 mRNA was restricted to the cytotrophoblast (27). In the same study, Ang-1 and Ang-2 were shown to be implicated in the regulation of trophoblast behavior through different mechanisms and to promote the growth and migration of trophoblasts *in vitro* (27). In particular, Ang-1 acts as a potent chemotactic factor for trophoblasts, whereas Ang-2 enhances trophoblast DNA synthesis and triggers nitric oxide release (27). In term placentas, Ang-1 mRNA expression is restricted to the perivascular tissues of the primary stem villi, supporting its role in vessel maturation. Ang-2 is expressed throughout the term villous core and maintains the plasticity of placental vessels (27). In addition, Tie-2 receptors are also expressed in trophoblasts and participate in the regulation of trophoblast behavior (27). The inconsistency observed between different studies may be due to the different gestational age at placental tissue sampling and the different methodologies used.

Experimental studies have also shed further light on the key role of angiopoietins in fetal viability and wellness. In a previous study, mice with null mutations in the Ang-1 gene died at day 12.5 of pregnancy and showed severe vascular abnormalities characterized by disturbance in endothelial/pericyte interactions (40). In another study, mice genetically deficient for Ang-2 showed complex lymphatic and vascular defects and the majority of the mice died two weeks post-natally (24). In turn, Geva *et al* (2005) used a novel murine model system and showed that *in utero* Ang-2 gene delivery impaired endothelial cell adhesiveness, leading to vascular leakiness with perivascular edema (41). Mice null for Tie-2 have been shown to exhibit severe vascular damage and cardiac abnormalities that cause embryonic lethality at approximately embryonic day 10.5 (42). A previous study by Wulff *et al* (2002) provided a detailed description of the spatial and temporal expression of Ang-1, Ang-2 and Tie-2 in the primate placenta and indicated that Ang-1/Tie-2 may support feto-placental vascular development and stabilization, whereas Ang-2/Tie-2 may remodel the maternal vasculature (43).

## 4. Angiopoietins in pregnancy complications

The majority of published studies on the role of angiogenic factors in the development of pregnancy disorders related to impaired placentation, such as PE and IUGR have focused on the expression profiles of VEGF, PlGF and its receptors. However, few studies have examined the expression pattern of angiopoietins in the above-mentioned pregnancy disorders.

PE affects approximately 2–7% of all pregnancies and is a major cause of maternal and perinatal morbidity and mortality (44). Although the pathogenesis of this condition is multifactorial, there is robust evidence indicating that PE is associated with impaired placentation and placental hypoxia related to an imbalance between pro-angiogenic and anti-angiogenic factors (4,45). Previous studies that have examined the placental expression of angiopoietins in pregnancies complicated by PE have presented conflicting results and have used rather small sample sizes (Table I). Zhang *et al* (2001) examined the placental tissue samples from nine women with PE at 31–40 weeks of pregnancy and demonstrated lower levels of Ang-2 mRNA expression compared to the control group (36). A similar study by Geva *et al* (2002) demonstrated that the placental Ang-1 and Tie-2 mRNA expression was not altered in five cases of PE with IUGR in the third trimester, whereas there was an increase in Ang-2 mRNA expression that did not reach statistical significance (35). In addition, Sung *et al* (2011) revealed no marked changes in the placental expression of Ang-1, Ang-2 and Tie-2 in placental samples from 19 preeclamptic pregnancies (46). In a recent study, the expression of Ang-2 was significantly increased in placentas obtained from pregnancies complicated by severe PE, whereas no change was observed for Ang-1 levels (47). In addition, no significant association was observed between Ang-1 and Ang-2 in the whole study group. However, the aforementioned study used a small study sample and the proportion of IUGR fetuses among the PE group was not reported. Similarly, a recent study by our group demonstrated an increase in the expression of Ang-2 in the PE and PE-IUGR group consisting of 58 pregnant women compared to the control group (48). To the best of our knowledge, this is the largest sample size in similar studies reported thus far. Our data also indicated that Ang-2 expression was even higher in the PE-IUGR group, suggesting a direct association of this angiogenic factor with the severity of the disease. Additionally, we detected the decreased expression of Ang-1 in PE, a trend that was not observed in the study by Han *et al* (47). Our analysis revealed a weak association between Ang-1 and Ang-2 in the PE-IUGR group and no significant correlation was observed between these parameters in the control and the PE group, in accordance with the study by Han *et al*. These findings are in line with those of earlier reports that support an excess of circulating levels of anti-angiogenic factors even before the clinical manifestation of PE (2,49). It is notable that Zhang *et al* (2001) examined how the oxygen tension in the placenta can influence the levels of angiopoietin expression and demonstrated that the Ang-1 mRNA level was not significantly altered by reduced oxygen, while a reduction in oxygen tension significantly increased the levels of Ang-2 mRNA through the activation of hypoxia-inducible factor-1α, leading to vessel instability and remodeling (36). This is important, as placenta hypoxia is an established factor of PE pathogenesis. Consistently, data from s study on bovine models demonstrated that hypoxia upregulated the expression of of Ang-2, but not Ang-1 mRNA expression (50).

The same inconsistency is also apparent in studies that have examined the maternal circulation levels of angiopoietins in pregnancies complicated by PE before or after the clinical manifestation of the disease. In cases with established PE, the majority of studies have reported lower circulating concentrations of Ang-2 (38,51,52) and Tie-2 (46,51,53) compared to the unaffected controls. However, Leinonen *et al* demonstrated that circulating maternal Ang-2 concentrations were elevated in the early midtrimester (16–20 weeks of gestation) in women that subsequently developed PE (37). These findings support the hypothesis that an excess of anti-angiogenic factors may be a predisposing factor for PE and may be apparent before the clinical onset of the disease. Moreover, the authors stated that the levels of Ang-2 were associated with the severity of the disease. Similarly, our recently published data demonstrated a higher placental expression of Ang-2 in pregnancies complicated by PE and IUGR, which is a distinct and more severe form of PE (48). A recent longitudinal study demonstrated the upregulation of Ang-2 levels and decreased Ang-1 levels in maternal serum in the whole gestational frame; in addition it was shown that the Ang-1/Ang-2 ratio at 25–28 gestational weeks may have predictive value for women who later developed PE (54). This is a very promising finding, as there is evidence supporting the ability of other angiogenic factors, such as PlGF to identify pathological pregnancy outcomes, such as IUGR and PE before clinical diagnosis with adequate sensitivity (55,56). At 11–13 weeks of gestation, the maternal serum concentration of Ang-2 has been reported to be either lower (57) or not altered in women who subsequently develop PE (58). Akolekar *et al* concomitantly examined the first trimester maternal serum levels of Ang-2 in pregnancies with gestational hypertension and found no significant changes compared to the control group (58). A recent study assessed Ang-1, Ang-2 and the Ang-1/Ang-2 ratio levels in the first trimester of pregnancy and the association with adverse pregnancy outcomes (small for gestational age, pre-term birth, PE, miscarriage after 10 weeks of gestation, and stillbirth) (59). According to the findings of the former study, low Ang-2 levels and a high Ang-1/Ang-2 ratio were related to an increased risk for most adverse pregnancy outcomes, but did not improve the prediction given by maternal and clinical factors alone (58). In support of this finding, first trimester maternal serum levels of Ang-1 were found to be elevated in women with PE and concomitant IUGR (37). As regards the studies on Tie-2 in maternal serum, it should be clarified that the measurable part is the circulating soluble Tie-2 receptor (sTie-2) fragment that is cleaved constitutively from the extracellular domain of membrane-bound Tie-2, binds both to Ang-1 and Ang-2 and inhibits angiopoietin-mediated Tie-2 activation. Moreover, data have suggested the existence of a novel metalloproteinase-dependent mechanism through which release of sTie-2 is induced by VEGF in endometrial endothelial cells and leads to a decrease in active receptors on the cell surface (60). As VEGF and Ang act through distinct tyrosine kinase receptors, this mechanism suggests that VEGF is involved in the regulation of Ang function indirectly through the proteolytic shedding of Tie-2.

The role of angiopoietins has only scarcely been investigated in pregnancies with fetal growth restriction, a condition that complicates 4–7% of live births in developed countries and is related to a spectrum of perinatal complications, including fetal morbidity and mortality. Placental insufficiency related to defective placental angiogenesis often represents the underlying cause of IUGR (61). In particular, placentas from pregnancies complicated IUGR are characterized by defective vascular transformation and terminal villus formation (3). At 11–13 weeks of gestation, Wang *et al* demonstrated that Ang-2 maternal serum levels were reduced in 13 women who subsequently developed IUGR compared to 23 unaffected controls (57). However, in that study, it was not defined whether IUGR was related to PE or could be classified as idiopathic. This is in contrast to the study by Leinonen *et al* that examined the circulating levels of Ang-1, Ang-2 and Tie-2 at 12–15 or 16–20 gestational weeks in 16 women with subsequent isolated IUGR compared to 59 healthy women served as controls (37). In that study, maternal serum Ang-2 concentrations were higher in women with subsequent IUGR only at 16–20 gestational weeks. Further studies are required to elucidate whether the involvement of these factors is a primary mechanism leading to the abnormal placental development or whether the altered expression of these angiogenic growth factors reflects other, as yet unidentified mechanisms. The altered placental expression of the angiopoietin/Tie-2 system has been observed in pregnancies complicated by placenta accreta and in women with recurrent abortion (62,63); however, these conditions are not within the scope of this review article.

## Conclusion

There is emerging evidence indicating that the angiopoietin/Tie-2 signaling system is essential for the proper reorganization of the placental vascular system even from the very first stages of pregnancy. In recent years, an increasing number of studies in the field of cancer research have not only highlighted the important role of angiopoietins in tumor progression, but have also attempted to implicate them as factors in the prognostic and therapeutic field (28,30,64,65). However, available information on the function of angiopoietins during pregnancy is still limited, as the main interest has been focused on the VEGF and PlGF systems. In addition, the majority of published studies are case-control studies that describe the observed alterations in the levels of angiopoietins in pathological pregnancies, but do not elucidate the aetiopathogenesis and the underlying mechanisms. Additional studies with sufficient sample sizes and strict criteria for the recruitment of study populations are required in order to draw a final conclusion as to the alteration profile of angiopoietins in pregnancies complicated by PE and/or IUGR. Moreover, the prognostic value of these factors as biomarkers for particular pathological conditions during pregnancy should also be investigated in prospective studies.

## Figures and Tables

**Table I:** List of studies that examined the levels of Ang-1, Ang-2 and Tie-2 in placental specimens from pregnancies complicated by PE and IUGR.

Authors/(Refs.)	Year	Sample size	Gestational frame in weeks	Pregnancy complication	Ang-1	Ang-2	Tie-2
Dunk *et al* (27)	2000	6	28–36	Severe IUGR		NS	
Zhang *et al* (36)	2001	9	31–40	PE		↓	
Geva *et al* (35)	2002	5	25–41	Severe PE		NS	
Sung *et al* (46)	2011	19		PE	NS	NS	NS
Han *et al* (47)	2012	16	28–41	Severe PE	NS	↑	
Kappou *et al* (48)	2014	28 PE + 30 PE/IUGR	34–39	PE PE + IUGR	↓	↑	↑

PE, preeclampsia; IUGR, intrauterine growth restriction; NS, not significant; The upward arrows indicate the upregulated expression and the downward arrows indicate the downregulated expression of Ang-1, Ang-2 and Tie-2 in the indicated studies.
